# A Trial Comparing Growing Rabbits Differing in 18 Generations of Selection for Growth Rate Reveals a Potential Lack of Effectiveness in the Genetic Selection Progress

**DOI:** 10.3390/ani13233625

**Published:** 2023-11-23

**Authors:** Pablo Jesús Marín-García, Eugenio Martínez-Paredes, Luis Ródenas, Lola Llobat, María Cambra-López, Enrique Blas, Juan José Pascual

**Affiliations:** 1 Departamento de Producción y Sanidad Animal, Salud Pública y Ciencia y Tecnología de los Alimentos, Facultad de Veterinaria, Universidad Cardenal Herrera-CEU, CEU Universities, 46022 Valencia, Spain; maria.llobatbordes@uchceu.es; 2 Institute of Animal Science and Technology, Universitat Politècnica de València, Camino de Vera s/n., 46022 Valencia, Spain; eumarpa@upvnet.upv.es (E.M.-P.); luiromar@dca.upv.es (L.R.); eblas@dca.upv.es (E.B.); jupascu@dca.upv.es (J.J.P.)

**Keywords:** growth rate, genetic selection, performance, rabbit, paternal line

## Abstract

**Simple Summary:**

There is evidence that the genetic progress in paternal rabbit lines is lower than expected due to animal- and environmental-related factors, as well as founding factors intrinsically linked to the effectiveness of the selection process. In response to this question, we conducted a trial to evaluate the response after 18 generations of selection for increased growth rate within a paternal line on rabbit performance. Our results indicate that there were no differences in the key indicators in selection for growth rate (average daily gain and feed conversion ratio) between both populations differing in the generation of selection. These findings indicate a lack of effectiveness in the genetic progress of paternal rabbit lines based on different works carried out with these same genetic lines and generations.

**Abstract:**

A total of 338 weaned rabbits (from the R line, selected for post-weaning growth rate) were used to evaluate the response to 18 generations of selection for increased growth rate on rabbit performance. Animals were obtained from two vitrified populations of the R line: R19V, belonging to the 18th generation (n = 165), and R37V, belonging to the 36th generation (n = 173), were allocated in individual and collective pens (178 and 160, respectively). A fattening trial was conducted from weaning (28 d of age until 63 d of age). During the trial, the body weight (BW), daily feed intake (DFI), average daily gain (ADG) and feed conversion ratio (FCR) were weekly monitored. Additionally, mortality and morbidity were daily registered. On days 49 to 53, an apparent faecal digestibility trial was also performed (12 animals per generation). Our results indicate that the generation of selection for growth rate did not affect mortality and morbidity. There were no differences in the diet digestibility according to the generation of selection. Regarding performance traits, R37V animals showed higher global BW (+6.7%; *p* = 0.0011) than R19V animals. R37V animals showed the same BW at weaning; however, R37V animals showed higher BW values in the last three weeks compared with R19V animals. Animals from the R37V generation also showed a higher DFI from 56 to 63 d of age (+12%; *p* = 0.0152) than R19V animals. However, there were no differences in global ADG and FCR between generations. These results indicate that the selection for growth rate in growing rabbits has slowed down, suggesting a lack of effectiveness in the genetic progress.

## 1. Introduction

A three-way crossbreeding scheme is widely used in rabbit meat production [1]. Females belonging to a farm are generally inseminated as a terminal sire with semen coming from paternal lines. As a result of this crossbreeding process, the growing rabbit is obtained. Feed conversion ratios (FCRs) are the most relevant economic traits in rabbit farming; the genetic progress focuses on improving these traits in maternal and paternal lines, respectively [2,3]. In the three-way crossbreeding scheme, the first cross is generally carried out with two different maternal lines aiming to take advantage of heterosis obtaining crossbred females with low inbreeding rates for the maternal traits [2,4,5]. These crossbred females are subsequently crossed with males belonging to the paternal lines. These lines are usually selected for average daily gain (ADG) since there exists a negative genetic correlationship between ADG and FCR [6].

Improving FCR is key to guaranteeing the sustainability of rabbit-rearing systems and an economically viable production for farmers. Therefore, the adequate selection of rabbit males from paternal lines can have a significant impact on the feed efficiency and competitiveness of commercial rabbits worldwide, as has been observed in other species [7,8].

Today, there is a widely used paternal line (R line; Universitat Politècnica de València) belonging to the two paternal lines; the first one was founded in 1976 with California rabbits reared by Valencian farmers and the second one was founded in 1981 with rabbits belonging to specialized paternal lines [2]. The selection criteria used for this line were individual ADG from 28 to 63 d of age. Therefore, the ADG of this line is high (on average 55 g/d) during the growing period. Paternal lines generally exhibit improved performance traits compared with maternal lines. For instance, García-Quirós et al. (2014) reported on av. +10.6 g/d for ADG and −0.25 g dry matter (DM) intake/g of ADG for FCR in paternal compared with maternal lines [9]. Despite this, some studies have shown that the genetic progress in paternal lines seems to be lower than that expected [10,11]. In these studies, the calculations expected based on superiority taking heterosis into account were performed, but they were carried out on growing rabbits from a three-way crossbreeding scheme differing both in the degree of selection for litter size at weaning and in the post-weaning growth rate.

We hypothesize that the response to genetic selection for an increased growth rate in growing rabbits may have been slowing down in recent years due to animal- and environmental-related factors, as well as founding factors intrinsically linked to the effectiveness of the selection process. Therefore, the main aim of this work was to evaluate the response after 18 generations of selection for an increased growth rate within a paternal line on rabbit performance to confirm this hypothesis. To this end, a trial was conducted to compare animal performance, mortality, morbidity and nutrient digestive efficiency in two populations of the R line differing in 18 generations of selection.

## 2. Materials and Methods

The experimental procedure was reviewed and approved by the Animal Welfare Ethics Committee of the Universitat Politècnica de València (code number 2015/VSC/PEA/00.061) and carried out following the recommendations of the European Group on Rabbit Nutrition and the regulations outlined in the Directive 2010/63/EU EEC and the Spanish Royal Decree 53/2013 on the protection of animals used for scientific purposes.

### 2.1. Animals

Animals used in this study belonged to the paternal line R, founded and selected at the Universitat Politècnica de València. The R line was founded in 1989, after two generations of random mating from a pool of animals of three commercial sire lines [2]. Line R is a synthetic line chosen by individual selection on the criterion of daily gain between weaning and slaughter (28 and 63 d).

Animals came from two populations of rabbit females and males differing in 18 generations of selection for the growth rate (R19V and R37V). The R19V population was rederived from 256 embryos of 25 donors belonging to eight different sire families of the 18th generation and vitrified in 2000. The R37V population was rederived from 301 embryos from 28 donors belonging to nine different sire families of the 36th generation, which were vitrified in 2015. Therefore, parents of both populations used in this study were stored as frozen embryos, thawed and transferred at the same time (2015) to rabbit females and males to obtain coetaneous populations [12].

**Figure 1:**
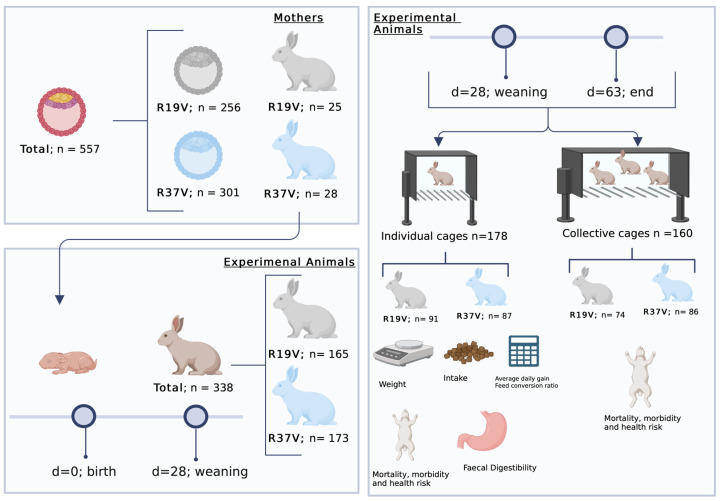
Summary of the experimental design used in this work. Created with BioRender.com (accessed on 9 September 2023).

After one generation of breeding within the population without selection to avoid the environmental maternal effect, young rabbit females and males from R19V and R37V were obtained and simultaneously compared (Figure 1). Detailed information concerning the cryopreservation and embryo transfer techniques used to obtain these populations is described in [13,14]. A total of 338 weaned rabbits (165 from R19V and 173 from R37V) were used.

### 2.2. Experimental Procedure

At 28 d of age, weaned rabbits were housed in individual and collective pens (178 rabbits housed in individual cages, 91 from R19V and 87 from R37V; and 160 rabbits were housed in collective cages, 74 from R19V and 86 from R37V). Individual pens (52 × 44 × 32 cm) were used to control performance traits and nutrient digestibility, mortality, and morbidity. The rest of the animals (n = 160) were located in 21 collective pens (152 × 44 × 32 cm), which were used to control mortality and morbidity.

All animals received the same commercial diet for growing rabbits, provided *ad libitum* throughout the experimental period (until day 63 of age) (Table 1). Individual body weight (BW) and daily feed intake (DFI) were recorded at 28, 35, 42, 49, 56 and 63 d of age. Performance traits were recorded weekly, while mortality and morbidity were recorded daily. The sanitary risk index was calculated as mortality + morbidity. The experimental procedure was carried out in nine consecutive batches, under controlled environmental conditions (animals were kept at 15 to 22 °C, with a photoperiod of 12 h of light and 12 h of darkness). The animals that died or that suffered from disease were extracted to calculate the productive parameters.

### 2.3. Digestibility Trial

A total of 24 growing rabbits (belonging to animals allocated in individual pens, 12 animals from each generation) were used to determine apparent nutrient faecal digestibility coefficients. At 42 d of age, randomly selected animals were housed in individual metabolic cages (52 × 44 × 32 cm^3^) and after a 7-day acclimatization period, a faecal digestibility trial was conducted [15]. From 49 to 53 d of age, feed consumption was registered, and faeces produced during the whole period were collected. Faeces were stored in identified plastic bags and frozen at −20 °C until analysis.

Feed and faeces were analyzed for dry matter, ashes, crude protein (CP), neutral detergent fibre (NDF), acid detergent fibre (ADF) and acid detergent lignin (ADL). The AOAC method for DM (934.01), CP (976.06) and ash (942.05) was used to analyze feed and faeces [16]. The NDF (assayed with a thermo-stable amylase and expressed exclusive of residual ash), ADF (expressed exclusive of residual ash) and ADL (determined by the solubilization of cellulose with sulphuric acid) were analyzed sequentially [17].

### 2.4. Statistical Analysis

For weekly BW, ADG, DFI and FCR, all data from each trait were studied using the following model:

$$y_{ijk}={GS}_{i}|{AGE}_{j}|B_{k}+p_{l}+e_{ijkl}$$

where $y_{ijk}$ represents one record of a given trait; ${GS}_{i}$ was the effect of the generation of selection (two levels: R19V and R37V); ${AGE}_{j}$ was the age of the growing rabbits (with six levels for BW: 28, 35, 42, 49, 56 and 63 d of age; and with five levels for the rest of the traits: week 1 [28–35 d of age], week 2 [35–42 d of age], week 3 [42–49 d of age], week 4 [49–56 d of age] and week 5 [56–63 d of age]); $B_{k}\;$ was the batch (with nine levels), $p_{l}$ and $e_{ijkl}$ were the permanent effect and the random residuals of the records, respectively.

This analysis was performed using the proc MIXED of SAS (2009), in a repeated measure design that considered the variation between animals and the covariation within them. Covariance structures were objectively compared using the most severe criteria (Schwarz Bayesian criterion) [18]. Apparent faecal digestibility coefficients were analyzed using the GLM model of SAS, including the generation as the only fixed effect (SAS, 2009).

Finally, the cumulative mortality, morbidity and sanitary risk index were analyzed by the GENMOD procedure (SAS, 2009) for each part of the experimental period. A binomial distribution was used for data and the link function was the logit transformation, ln [μ/(1 − μ)], where μ was the mean value including the generation of selection as the only explanatory variable. All data were presented as least-squares means, which were compared using a protected t-test, differences being considered as significant at *p* < 0.05.

## 3. Results

Mortality was on average 30% during the trial. No significant differences were observed either due to the genetic line (31 and 29% for R19V and R37V, respectively) or due to the type of accommodation (28% and 31% in individual and collective pens, respectively). The generation of selection for the growth rate did not significantly affect the cumulative mortality and morbidity (average morbidity equal to 34%). Additionally, no statistically significant differences were observed in the apparent nutrient faecal digestibility coefficients between generations (Table 2).

Table 3 shows the global performance of growing rabbits differing in the selection degree for the growth rate from 28 to 63 d of age. Our data show that the average initial BW was 504 g, DFI was 123 g/d, ADG was 49.3 g/d and FCR equaled 2.52. The R37V animals showed higher BW at 63 d of age (+3.7%; *p* < 0.01) and slightly higher global DFI values (+3.2%; *p* < 0.10) than the R19V animals. There were no statistically significant differences in global ADG and FCR between the two generations.

Regarding performance traits, R37V animals showed higher global BW (+6.7%; *p* = 0.0011) than R19V animals. R37V animals showed the same BW at weaning; however, R37V animals showed higher BW values than R19V animals in the last three weeks. Animals from the R37V generation also showed the same ADG throughout the whole experimental period and a higher DFI from 56 to 63 d of age (+12%; *p* = 0.0152) than R19V animals.

**Figure 2:**
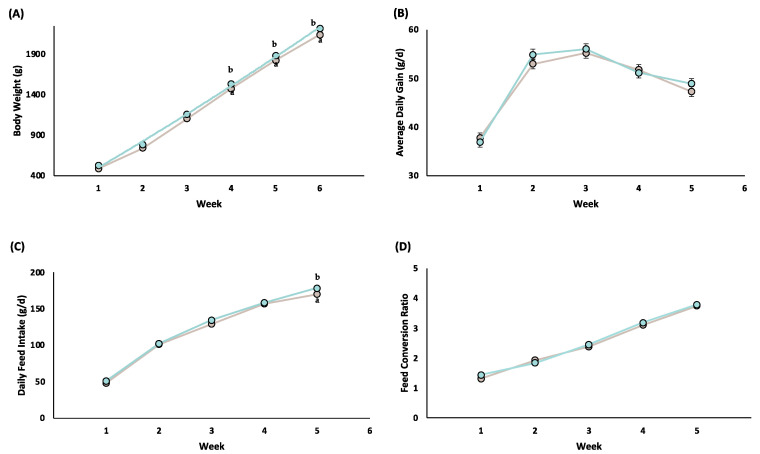
Effect of the generation of selection for growth rate (R19V 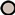 and R37V 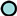) on body weight (**A**), average daily gain (**B**), daily feed intake (**C**) and feed conversion ratio (**D**) in function of the time. Week 1 (28–35 d); week 2 (35–42 d); week 3 (42–49 d); week 4 (49–56 d); week 5 (56–63 d). ^a,b^ Means within a row with a different letter were significantly different at *p* < 0.05. Only performed on healthy animals.

Figure 2 shows the effect of the generation of selection for growth rate on the performance of the animals in the different periods. The R37V animals showed a higher BW throughout the whole trial compared with the R19V animals (on av. +4.2%; *p* < 0.05), although differences were only significant from 49 days of age onwards. The DFI was also slightly higher in R37V animals during the last week of the growing period, week 5 (+4.9%; *p* < 0.01).

## 4. Discussion

The main aim of this work was to determine whether the selection for the post-weaning growth rate could have affected the growth performance and nutrient digestive efficiency in growing rabbits. This condition could be useful for verifying the adequate progress of the genetic selection by this criterion in this species. To achieve this goal, we developed two populations using cryopreserved material from two different generations of the same line, managed under the same conditions, which were concurrently compared. In this way, the differences between populations would be solely attributable to the generation of selection by the growth rate, excluding the other environmental factors.

Global mortality (30%) observed during the trial was higher than that expected for this same line in the fattening period. The trial was carried out on an experimental farm where epizootic rabbit enteropathy outbreaks are frequent; it should be mentioned that the number of animals used could represent a limit regarding the effect of generation on mortality and morbidity. We used unmedicated feed because one of the objectives was to evaluate the sensitivity to suffering from digestive diseases in the function of the generation, in a line which is especially sensitive to these diseases (on av. 24% mortality). However, global morbidity (4%) was slightly lower than that expected (on av. 11.3% under the same conditions) [9,19,20]. As a result, the obtained sanitary risk index (37%) was comparable to other reported values in the literature for this same line (on av. 35%) [9,19,20,21].

In spite of the fact that post-weaning mortality may depend on the genotype of the animal [22], we observed no differences due to the effect of the generation of selection for the growth rate in mortality, morbidity and health risk. These data are in accordance with other studies where no differences were observed due to the genetic type comparing other lines [23] or even when comparing this same line with a maternal line (H line) [9]. These results seem to indicate that the greater sensitivity of a certain genetic line to suffering from particular diseases is not related to the genetic selection criterion used. Instead, the higher mortality values observed for this paternal line compared to other maternal lines [7] seem to be more associated with foundational aspects or the management of the populations during the selection process throughout the years.

The obtained coefficients of apparent faecal digestibility agree with those obtained for this same genetic type and age [19,20,24,25]. We observed no statistically significant differences in nutrient digestive efficiency between generations. No significant differences in apparent faecal digestibility coefficients of the main nutrients in rabbit females coming from a maternal line (V line) differentiating in 20 generations of selection for litter size at weaning (16 vs. 36 generations [26]) were reported. Furthermore, no differences in DM apparent faecal digestibility between the two different genetic lines (V vs. LP) in growing rabbits were seen [27]. These results agree with what happens in prolific species [28,29], where there is generally no effect on the digestive efficiency of the main nutrients associated with the breed or commercial line used. This indicates that the selection process does not change digestive efficiency.

Average BW, ADG and DFI values obtained in this trial were slightly lower than those obtained for this line in previous trials on the same experimental farm [19,20,25,30,31]. In general, the results of this study seem to indicate that selection for the growth rate during the growing period has not been effective in this line. No significant differences in ADG and FCR were observed between animals from generations 19 and 37. Although the final BW at 63 days of age was higher in most selected animals, the selection program only led to an ADG of +0.6 g/d after 18 generations of selection (+0.032 g/d per generation). Although there are no reliable estimates of the selection in this line, previous studies [11,32,33] have proposed it to be around +0.5 g/day per generation. This means that the expected superiority of the R37V should have been of +9 g/d after 18 generations, which is considerably superior to the result obtained. It has been proven that the genetic progress in a studied line seems to be lower than that expected from the heritability of this character (in this case, the phenotypical improvement observed was 33% of that expected in the last years of selection [10,11]). In fact, no significant differences were observed in the GR of any of the body components analyzed, studied in this same paternal line but with low generational differences [34,35]. It would be interesting to also note the effect observed when these productive data are analyzed weekly. Observations of ADG and DFI during the first week indicate values that are lower by 15% and 35%, respectively, compared to the recent study on the same genetic line. While these disparities did not reach statistical significance among the experimental groups in our current research, it is essential to consider that the animals started with a lower initial weight, a factor that was, however, compensated for by the end of the growth period. The differences observed may be attributed to variations in initial weights, emphasizing the importance of accounting for such factors in the interpretation of the results [25]. The differences observed in the final section of the bait respond to the same period where significant differences were observed in some of these same productive traits when feeds with different levels of protein were analyzed in animals of this same genetic line (but all from the RV37 generation). This could indicate that this genetic type is especially susceptible to the final stages of growth, showing behavior like that at the beginning [19].

The common criterion for selecting rabbits in paternal lines has often been individual selection by ADG. This approach is favored due to its ease of recording and a moderate heritability ranging from 0.15 to 0.18, along with a positive genetic correlation with the FCR [36,37]. In this context, the response to selection for ADG has been assessed through comparisons with a control population [23,38] or through divergent selection strategies. The observed response over generations has shown a variation ranging from 0.56 g/d to 6 g/day [36,39]. In summary, our data reveal a potential slowdown in the genetic selection progress of growing rabbits of this paternal line. This probable lack of effectiveness in the genetic progress could be due to (i) their reaching a biological threshold at the genetic selection level, (ii) problems related with a reduced number of available animals and the consequent lower selection pressure, or (iii) nutritional deficits in current commercial diets when these are fed to animals with a high growth rate. In this regard, the first hypothetical cause (i) seems not to be plausible because many studies have indicated that there exists enough genetic variability on feed efficiency to improve the genetic advance in this line [1]. In fact, today it is possible to show animals with an extremely high GR (>87 g/d). Regarding the second hypothetical cause (ii), reproductive traits are significantly lower in paternal lines, as the studied line. There are some works where females belonging to this studied line revealed a lower litter size [40], ovulation rate [5,41], fertility [39] and litter survival [7] compared with females belonging to maternal lines. It is also interesting to explain that if the offspring are not very numerous (available animals), the response to selection is conditioned. This fact could be accentuated by the high mortality and morbidity having an influence on the results. This hypothesis seems more plausible than the alternative since there are works that suggest that rederivation by cryopreservation of this paternal line (R line) of rabbits indicates exhaustion of selection for this trait after all generations [40,41,42].

Irrespective of this fact, the third cause (iii)—the nutritional cause—could have a relationship with a potential lack of genetic progress or expression of the genetic potential of animals. Animals with a higher ADG would also have higher nutrient requirements, especially in limiting amino acids. This could be explained because there is a positive relationship between growth and protein requirements. In this regard, it has been reported that animals of the R line have different amino acid requirements and show a different growth potential when the levels of some amino acids are modified [19,20]. In this case, we have used a commercial diet aiming to establish the effects of selection in situations like those in which the aforementioned genetic selection occurs. This potential nutritional deficit, when high-growth-rate rabbits are fed current commercial diets for growing rabbits, could probably elucidate a portion of the lack of effectiveness of the genetic selection observed in this work.

Different trials on these animals suggest that the improvement in this line has been positive in the first generations but has stagnated; in a work that was used for these same generations, it was concluded that after 37 generations of selection, this trait seems exhausted. The results of this work are precisely along those lines but extending these observations to a productive stage that had not yet been analyzed, the growing rabbits, a very important productive group since it is precisely in this physiological stage that we expect the productive improvements in this genetic line.

## 5. Conclusions

Based on our findings, we can conclude that selection for the growth rate in a paternal line of rabbits, by comparing two populations differing in 18 generations of selection, did not affect animal growth performance, health status (mortality and morbidity) and digestive nutrient efficiency as expected. Although the final body weight of the R37V animals was increased, there were no significant differences in either the ADG or FCR between both generations, the main aim of the genetic program in this line. Obtained results suggest a potential lack of effectiveness in the genetic selection for post-weaning daily weight gain after 37 generations in the line used.

## Figures and Tables

**Table 1:** Ingredients (g/kg) and chemical composition (g/kg dry matter) of the experimental diet.

Ingredients	
Wheat bran	303
Straw	177
Sunflower meal	198
Alfalfa hay	105
Beet pulp	121
Barley	20
Beet molasses	29
Flour cookies	30
Palm oil	3.5
Sodium chloride	4.0
Calcium carbonate	4.5
L-Threonine	0.6
DL-Methionine	0.5
Vitamin-mineral mix ^1^	3.0
Chemical composition	
Dry matter (DM; g/kg)	897
Ashes	76
Crude protein	153
Crude fibre	195
Ether extract	26.9
Neutral detergent fibre	365
Acid detergent fibre	204
Acid detergent lignin	42.2
Starch	156

^1^ Contains per kg of feed: vitamin A: 8375 IU; vitamin D3: 750 IU; vitamin E: 20 mg; vitamin K3: 1 mg; vitamin B1: 1 mg; vitamin B2: 2 mg; vitamin B6: 1 mg; nicotinic acid: 20 mg; choline chloride: 250 mg; magnesium: 290 mg; manganese: 20 mg; zinc: 60 mg; iodine: 1.25 mg; iron: 26 mg; copper: 10 mg; cobalt: 0.7 mg; butyl hydroxylanysole and ethoxiquin mixture: 4 mg.

**Table 2:** Apparent faecal digestibility coefficients of nutrients (lsmean ± standard error) according to the generation of selection (R19V and R37V), performed on healthy animals.

		Generation of Selection				
	No.	R19V		R37V		*p*-Value
No. of observations at 63 d of age		12		12		
Dry matter (%):	24	56.42	±0.82	57.28	±0.90	0.5397
Ashes (%)	24	7.52	±1.68	7.74	±0.60	0.7435
Crude protein (%)	24	74.43	±0.82	74.11	±0.84	0.7826
Neutral detergent fibre (%)	24	69.63	±1.87	68.82	±1.62	0.2697
Acid detergent fibre (%)	24	40.51	±0.41	39.98	±0.42	0.3742
Acid detergent lignin (%)	24	9.15	±0.23	9.03	±0.23	0.7334

Population rederived from embryos of line R vitrified at 19 (R19V) or 37 (R37V) generation of selection for growth rate after weaning.

**Table 3:** Growing rabbit global performance traits during the whole growing period (28 to 63 d) in function of the generation of selection for growth rate (lsmeans ± standard error), only performed on healthy animals.

		Generation of Selection				
	No.	R19V		R37V		*p*-Value
No. of observations at 63 d of age		83		80		
Live weight at 28 d (g)	163	484.9	±30.4	523.5	±28.9	0.1818
Daily feed intake (g/d)	163	121.1	±2.5	125.0	±2.3	0.0875
Average daily gain (g/d)	163	49.00	±0.7132	49.57	±0.67	0.3745
Feed conversion ratio	163	2.496	±0.041	2.543	±0.038	0.2105
Live weight at 63 d (g)	163	2139	±30.4	2218	±28.6	0.0062
Mortality (%)	163		31		29	0.6578

Population rederived from embryos of line R vitrified at 19 (R19V) or 37 (R37V) generation of selection for growth rate after weaning. Measured in healthy animals.

## Data Availability

Data are contained within the article. The datasets of the current study are available from the corresponding author upon reasonable request.
